# Health protection policies for digital platform and low wage workers

**DOI:** 10.1093/eurpub/ckac129.375

**Published:** 2022-10-25

**Authors:** E MacEachen

**Affiliations:** School of Public Health Sciences, University of Waterloo, Waterloo, Canada

## Abstract

**Background:**

In the context of the COVID-19 pandemic, shifting employment and occupational health social protections of low-wage and self-employed digital platform workers are described and compared. Specifically, we examine how, across advanced economy countries, laws, policies, and collective agreements protected the health of low wage (e.g., service workers) and digital platform workers (usually classified as self-employed) including during the first three waves (2019-2021) of the COVID-19 pandemic. The overall goal is to inspire conversation, comment, critique and new research questions to tackle the issue of the employment, work and health of low wage workers and self-employed digital platform workers.

**Methods:**

Taking a comparative focus on eight advanced economy countries, this paper identifies legal efforts to address employment misclassification and challenges related to employee definitions that vary by the legal act. Debates about minimum wage and occupational health and safety standards as these relate to worker well being are considered. Finally, we discuss promising changes introduced during the COVID-19 pandemic that protect the health of low-wage and self-employed workers.

**Results:**

Overall, we describe an ongoing “haves” and a “haves not” divide, with on the one extreme, traditional job arrangements with good work-and-health social protections and, on the other extreme, low-wage and self-employed digital platform workers who are mostly left out of schemes. However, during the pandemic small and often temporary gains occurred and are discussed.

**Conclusions:**

In the context of an evolving social contract during the COVID-19 pandemic, this paper provides views on avenues for policy reform and research from employment and occupational health specialists across eight advanced economy countries.

